# Early biomarkers for prediction of severe manifestations of dengue fever: a systematic review and a meta-analysis

**DOI:** 10.1038/s41598-023-44559-9

**Published:** 2023-10-14

**Authors:** Samaneh Moallemi, Andrew R. Lloyd, Chaturaka Rodrigo

**Affiliations:** 1 School of Biomedical Sciences, Faculty of Medicine and Health, UNSW Sydney, Sydney, NSW 2052 Australia; 2 Viral Immunology Systems Program, Kirby Institute, UNSW Sydney, Sydney, NSW 2052 Australia

**Keywords:** Biomarkers, Predictive markers

## Abstract

Early identification of dengue patients at risk of adverse outcomes is important to prevent hospital overcrowding in low- to middle- income countries during epidemics. We performed a systematic review to identify which biomarkers measured in first 96 h of fever could predict dengue haemorrhagic fever (DHF, World Health Organization 1997 clinical classification) or severe dengue (SD, WHO 2009, clinical classification). PubMed, Scopus, CINAHL, Web of Science, and EMBASE databases were searched for prospective cohort and nested case–control studies published from 1997 to Feb 27, 2022. The protocol for the study was registered in PROSPERO (ID: CRD42021230053). After screening 6747 publications, and analysing 37 eligible studies reporting on 5925 patients, elevated C-reactive protein, aspartate aminotransferase, interleukin-8 and decreased albumin levels were strongly associated with dengue haemorrhagic fever (by meta-analyses of multiple studies, p < 0.05), while elevated vascular cell adhesion protein 1, syndecan-1, aspartate aminotransferase and C-reactive protein levels were strongly associated with severe dengue (by meta-analyses of multiple studies, p < 0.05). Further 44 and 28 biomarkers were associated with the risk of DHF and SD respectively, but only in a single study. The meta-analyses suggest the importance of early acute inflammation with hepatic involvement in determining the subsequent course of illness in dengue.

## Introduction

Dengue infection, despite having a case fatality rate of less than 1%^1^, imposes a significant burden on healthcare resources due to the very large number of infections reported annually (an estimated 50–200 million cases annually worldwide)^2^. Active disease transmission occurs in 128 countries, and most of the disease burden is in tropical, low- and middle-income countries^2^. The incidence of dengue in these countries is typically seasonal, with a large proportion of cases being reported within the space of a few months during and after the wet season, overwhelming limited healthcare resources and putting both dengue and non-dengue patients at risk of preventable adverse outcomes. In dengue, adverse outcomes mostly occur in a subgroup of patients experiencing plasma leakage (increased capillary permeability with fluid extravasation), and this typically occurs after the first 96 h, around days 5–7 of fever. Thus, a system to predict those at risk of severe disease early in the infection (within first 96 h of fever) could prevent unnecessary hospital admissions and enable early discharges, ensuring equitable distribution of resources to patients who will most likely benefit from hospital admission. Individual studies have identified demographic, clinical, genetic, virological and immunological risk predictors for severe disease,^3^ but with often conflicting findings across studies.

Systematic reviews of these studies to identify commonly observed trends in risk predictors for severe dengue while adjusting for confounders across studies may be informative. Unfortunately, the quality of systematic reviews on dengue are affected by the heterogeneity of study designs and varied outcome definitions. The ideal study design for predicting risk factors for adverse outcomes in dengue is a prospective cohort study (or a nested case–control study within a prospective cohort) collecting data and samples for analysis within the first 96 h of the illness when the likelihood of serious adverse outcomes is low. Patients should then be followed prospectively to systematically record the occurrence of adverse outcomes according to criteria agreed a priori. Retrospective studies are unlikely to achieve the precision and reliability of prospective study designs because when outcomes (e.g., severe dengue, plasma leakage) are extracted from hospital records, the inter-observer bias (of those originally recording the observation) is not controlled. Furthermore, outcomes such as plasma leakage can be determined in several ways (haematocrit, ultrasonography or both) and uniformity of outcome definition cannot be guaranteed retrospectively as can be done by a study protocol for a prospective study. Even with prospective studies, the variation in outcome definitions reported in individual studies according to either the 1997 or 2009 World Health Organization clinical disease classifications^4,5^, as well as other classification systems (i.e., presence or absence of plasma leakage)^6^ pose a problem for systematic reviews as these outcomes are not directly comparable to allow inclusion in a meta-analysis even if reporting on the same risk factor.

The aim of this systematic review was to identify host biomarkers, measured within the first 96 h of onset of disease, associated with severe disease manifestations recorded later in the course of illness. Only prospective cohort studies and nested case control studies were considered to preserve the quality of evidence. There are few previous systematic reviews which have examined a similar research question on dengue. However, these focussed only on children^7^, a single type of biomarker (e.g., lipoproteins)^8^, included retrospective or cross-sectional case control studies affecting the quality of evidence, or included measurements beyond the first 96 h of fever, thus diminishing the clinical relevance of evidence^9^. Some other comprehensive recent systematic reviews on risk associations for severe dengue do not address the topic of biomarkers in-depth^3^. The last review on biomarkers in dengue with comparable scientific rigor had a search date in 2019^10^, and several new eligible studies were published in the interval.

## Methods

### Inclusion criteria

Prospective cohort studies and nested case control studies, which recruited laboratory confirmed dengue patients (adults and children) within the first 96 h from the onset of illness and compared concentrations of biomarkers assessed within this time window, as predictors of prospectively defined disease severity categories were eligible. The definition of biomarkers in this review included peptides or proteins, metabolites, electrolytes, and lipoproteins measured in plasma/serum, urine or other body fluids (e.g., saliva). The adverse outcomes were classified according to three systems commonly used in dengue clinical research: dengue fever vs. dengue haemorrhagic fever grades I-IV^5^, dengue fever with or without warning signs vs. severe dengue^4^, and absence vs. presence of plasma leakage.

Retrospective and cross-sectional case control studies, studies reporting secondary data (systematic reviews), those not reporting biomarker measurements within the first 96 h of fever, those only reporting on demographic, genetic, transcriptomic, viral, haematological (cell counts), and clinical (signs and symptoms) risk predictors, and those only involving animal or in vitro studies were excluded. The full list of exclusion criteria is provided in Supplementary Table S1.

### Search strategy

We searched PubMed, Scopus, CINAHL, Web of Science and EMBASE using the keywords "dengue" and ("plasma leakage" OR "critical phase" OR "hemorrhag*" OR "DHF" OR shock), and ("risk" OR "biomarkers" OR "predict" OR "prognos*") in abstract, title and keywords, without language restrictions (Table 1). Bibliographies of eligible articles and that of previous similar systematic reviews were searched manually as a secondary search. Only articles published after 1997 were considered to maintain recency of evidence and to avoid conflicts in outcome definitions due to older clinical classifications. The last date of the search was 27th February 2022. Authors of eligible studies were contacted for missing information and both published and unpublished data were included.

**Table 1:** Search strategy and results (Last date of search: 27th February 2022).

Database	Search strategy	Number of hits
PUBMED	(Dengue[Title/Abstract] AND (DHF[Title/Abstract] OR plasma leakage[Title/Abstract] OR critical phase[Title/Abstract] OR shock[Title/Abstract] OR hemorrhag*[Title/Abstract]) AND (risk[Title/Abstract] OR biomarkers[Title/Abstract] OR predict*[Title/Abstract] OR Prognos*[Title/Abstract] OR Sever*[Title/Abstract]))	1020
Scopus	(TITLE-ABS-KEY (*dengue* AND (*dhf* OR *"plasma leakage"* OR *"critical phase"* OR *shock* OR hemorrhag*) AND (*risk* OR *biomarkers* OR predict* OR prognos* OR sever*))	2130
Web of Science	(TITLE-ABS-KEY (dengue AND (*dhf* OR *"plasma leakage"* OR *"critical phase"* OR *shock* OR hemorrhag*) AND (*risk* OR *biomarkers* OR predict* OR prognos* OR sever*))	1980
CINAHL	(TITLE-ABS-KEY (dengue AND (*dhf* OR *"plasma leakage"* OR *"critical phase"* OR *shock* OR hemorrhag*) AND (*risk* OR *biomarkers* OR predict* OR prognos* OR sever*))	142
EMBASE	(TITLE-ABS-KEY (dengue AND (*dhf* OR *"plasma leakage"* OR *"critical phase"* OR *shock* OR hemorrhag*) AND (*risk* OR *biomarkers* OR predict* OR prognos* OR sever*))	1475

### Data collection and analysis

#### Study selection

Two authors (SM and CR) independently screened abstracts and identified articles for full-text review. Any disagreements were resolved by consensus and after consulting with the third author (AL). In addition to exclusion criteria given in Supplementary Table S1, some studies initially considered eligible were later removed after full-text review and these are listed in the characteristics of excluded tables (Supplementary Table S2).

#### Data extraction

From the included studies (Table 2 and Supplementary File S1), the following data were extracted: study population and time window of recruitment, country of origin, clinical classification system of severity, method of dengue diagnosis, method of biomarker measurement, sample size in each disease severity category, name and concentrations of biomarkers assessed. Initial data extraction was done by SM using Microsoft Excel 365 and all entries were independently re-checked by CR. When studies reported biomarker levels for the entire duration of the illness, only data reported for the first 96 h of fever (or a time window within that period) were extracted. If the biomarker concentrations and their variance were only reported in a figure (not in text or within supplementary material), an enhanced version of the figure was printed out and measures of central tendency (e.g., mean, median) and the measure of dispersion (e.g., standard deviation or interquartile range) was estimated from the y axis of the graph. Data reported as relative concentrations or fold changes (instead of absolute values) were only considered for narrative descriptions. For meta-analyses, median and interquartile ranges were converted to mean and standard deviation using an approximation method described by Wan et al.^11^. Standard error of mean and confidence intervals of mean were converted to standard deviation using methods described in the Cochrane handbook^12^.

**Table 2:** Characteristics of included studies.

Study	Method of confirmation of dengue diagnosis	Country	Recruitment time	Clinical classification system	Number of patients	Included in meta-analysis? (yes/no)
Biswas, 2015^13^	RT-PCR, viral isolation, IgM ELISA	Nicaragua	2005–2013	Both 1997 and 2009	789	Yes
Chaiyaratana, 2008^14^	IgM, IgG ELISA	Thailand	2002–2005	1997	177	No
Conroy, 2015^15^	IgM ELISA, viral isolation	Colombia	Not mentioned	1997	111	Yes
Cui, 2016^16^	RT-PCR, NS1 detection	Singapore	Not mentioned	1997	116	Yes
Han, 2019^17^	IgM, NS1 Elisa,	China	2016	1997	36	No
Hapugaswatta, 2021^18^	NS1 rapid test	Sri Lanka	2015–17	2009	127	No
Houghton, 2010^19^	RT-PCR	Colombia	2005–2006	1997	38	No
Koraka, 2004^20^	IgM, IgG ELISA	Indonesia	1995–96	1997	32	No
Kularatnam, 2019^21^	IgM ELISA	Sri Lanka	2013–14	1997	130	No
Kumar, 2012^22^	RT-PCR	Singapore	2005–06	1997	58	Yes
Lam, 2020^23^	RT-PCR, NS1 antigen, IgM assays	Vietnam	2013–15	2009	75	Yes
Laur, 1998^24^	RT-PCR, IgM assay,	Tahiti	1996–97	1997	52	No
Liao, 2015^25^	Antibody tests, virus isolation in C6/36 mosquito cells	China	2009	2009	51	No
Lin, 2019^26^	NS1 Ag STRIP, RT-PCR, and serology	Taiwan	2014—2016	2009	108	Yes
Mapalagamage, 2018^27^	IgM ELISA, rapid immunochromatography test (NS1)	Sri Lanka	2014–16	2009	138	No
Mapalagamage, 2020^28^	IgM ELISA/rapid chromatographic detection NS1	Sri Lanka	2015–16	1997	80	Yes
Mariappan, 2021^29^	NS1 ELISA, IgG, IgM ELISA	India	Not mentioned	2009	47	Yes
Nhi, 2016^30^	serologic assays, virus isolation, RT-PCR	Vietnam	2011–12	2009	63	No
Pang, 2016^31^	RT-PCR, IgM, IgG ELISA	Singapore	2005–08/2009–12	2009	158	No
Rathore, 2020^32^	NS1 ELISA, RT-PCR	Sri Lanka	Not mentioned	1997	84	No
Saniathi, 2021^33^	Not mentioned	Indonesia	2015–16	1997	80	Yes
Sigera, 2019^34^	RT-PCR	Sri Lanka	2017–20	2009	32	Yes
Silva, 2021^35^	NS1 antigen test,RT-qPCR	Sri Lanka	2018–19	2009	120	No
Suwarto, 2017^36^	RT-PCR, IgG, IgM ELISA	Indonesia	2013–15	1997	103	Yes
van de Weg, 2014^37^	RT-PCR, ELISA	Brazil	2011–12 Aruba/2010Brazil	2009	44	No
Villamor, 2017^38^	NS1, RT-PCRIgM ELISA	Colombia	2003–09/2009–10	1997	345	Yes
Villamor, 2018^39^	NS1, IgM ELISA, RT-PCR	Colombia	2003–09/2009–10	1997	344	No
Villar-Centeno, 2008^40^	viral isolation, IgM assays	Colombia	Not mentioned	1997	201	Yes
Vuong, 2021^41^	NS1 IgM ELISA, RT-PCR, viral isolation	Vietnam, El Salvador, Cambodia, Malaysia	2011–15	2009	281	Yes
Vuong, 2020^42^	NS1 ELISA, RT-PCR	Vietnam, El Salvador, Cambodia, Malaysia	2011–15	2009	1117	Yes
Yacoub, 2017^43^	NS1 ELISA, RT-PCR	Vietnam	2013–15	2009	293	No
Yacoub, 2016^44^	RT-PCR, NS1 antigen or DENV IgM, IgG assays	Vietnam	2013–14	2009	132	Yes
Yamanaka, 2013^45^	RT-PCR-NS1, IgM, IgG ELISA	Indonesia	2008–10	1997	130	Yes
Zain, 2017^46^	IgM IgG ELISA for serotyping only	Indonesia	Not mentioned	2009	42	No
Chandrashekhar, 2019^47^	IgG, IgM ELISA, NS1	India	2016–2018	2009	77	No
Fernando, 2016^48^	IgG, IgM, NS1 ELISA,	Sri Lanka	2015	2009	30	Yes
Low, 2018^49^	NS1 ELISA, RT-PCR	Malaysia	2016–2017	2009	82	No

#### Quality assessment

Multiple quality assessment tools were considered for this study and the closest which met the requirements for this meta-analysis was the tool developed by Wirsching et al.^50^ to assess the quality of biomarker based cross-sectional studies (BIOCROSS). A version with minor modifications was developed to fit prospective cohort studies (Supplementary Table 3). The quality of studies was independently evaluated by CR and SM. The modified BIOCROSS tool includes ten items covering five domains: ‘Study rationale’ ‘Design/Methods’ ‘Data analysis’, ‘Data interpretation’, and ‘Biomarker measurement’ with a maximum score of 20. An arbitrary cut-off score of 14 (70% of total score) was used to define “Tier 1” studies of high quality. All other studies were assigned to “Tier 2”. Any disagreements were resolved by consensus of all authors. Study quality assessment was also recorded on a Microsoft Excel 365 worksheet.

#### Data analysis

Studies were combined in a meta-analysis only if they were comparable both for the biomarker assessed and the outcomes reported (e.g., WHO 1997 or 2009 clinical guidelines). If one study reported daily variation of biomarkers and another the mean value over many days within the 96-h time window, daily mean and standard deviation values were combined according to the Cochrane-recommended formula for meta-analysis to have the overall mean and standard deviation representative of the 96 h period^51^. Some studies combined all 4 grades of dengue haemorrhagic fever (DHF I-IV) together, while others reported biomarker levels separately. To avoid heterogeneity, such studies were not combined. Meta-analyses were done with Review Manager version 5.4.1. (Cochrane Collaboration, 2020) using a fixed effect model. A p-value < 0.05 was considered statistically significant.

#### Assessment of heterogeneity

The strict selection criteria mentioned above was expected to reduce study heterogeneity in a meta-analysis, but we also assessed heterogeneity post-meta-analyses using the I^2^ statistic (> 70% for high heterogeneity)^51^. In the case of high heterogeneity, the results were re-analysed with a random effects model.

#### Assessment of reporting bias

This was performed only if more than 10 studies were eligible to be combined in a single meta-analysis.

#### Subgroup and sensitivity analysis

A subgroup analysis was not done. However, a sensitivity analysis undertaken by combining following categories as “severe disease”: severe dengue according to 2009 WHO classification, all categories of DHF according to 1997 WHO classification, and plasma leakage when this outcome alone was reported. All other patients were categorised as “non-severe disease”. This approach has been used previously in a meta-analysis by Sangkaew et al.^3^.

### Study protocol

The protocol for this systematic review was registered in PROSPERO (https://www.crd.york.ac.uk/prospero/, ID: CRD42021230053).

## Results

**Figure 1:**
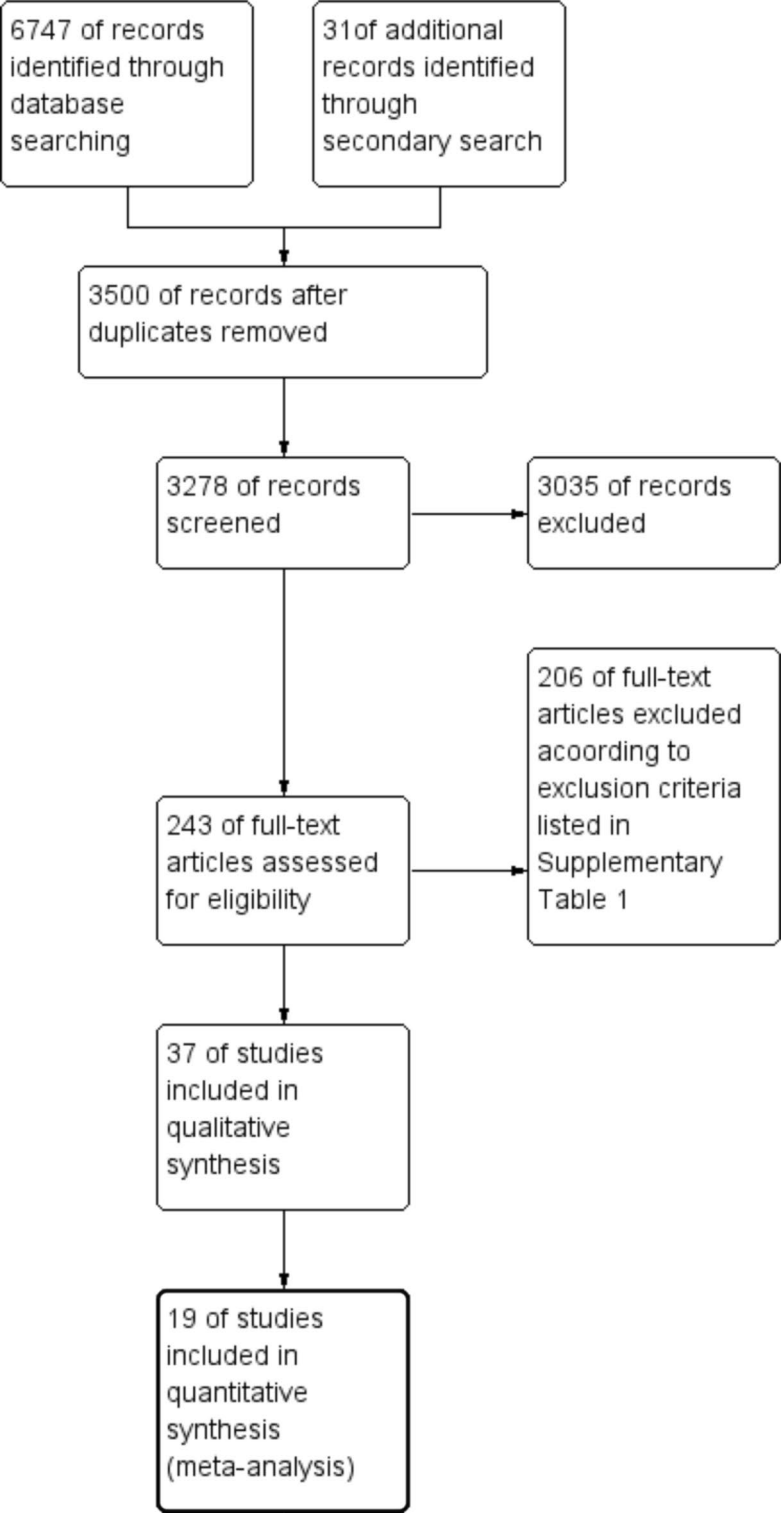
PRISMA flow diagram of study selection.

Thirty-seven studies that included 5925 patients met the inclusion criteria. Of these, 17 studies reported outcomes according to the WHO 1997 clinical classification (DF vs. DHF)^14–17,19–22,24,28,32,33,36,38–40,45^, and 17 reported outcomes according to the WHO 2009 clinical classification^18,25–27,29–31,34,35,37,41,42,44,46–49^, One study reported outcomes according to both WHO clinical classifications^13^, and two reported the severity outcome as the presence or absence of plasma leakage^23^. Since the latter group had only a limited number of studies, presence of plasma leakage in this group was considered as similar to DHF in the 1997 clinical classification for the purpose of the meta-analyses. Thirteen studies initially considered as eligible were later excluded (see excluded studies table, Supplementary Table S2). The eligible studies recruited patients between 1995 and 2019, mostly from South and Southeast Asian countries and Latin American countries. 19 studies were combined in a meta-analysis as two or more reported on the same biomarkers and outcomes (Fig. 1). Seven studies, despite measuring a biomarker that had been assessed in one or more other studies, did not report variation of measurements or the units of measurements^14,20,21,24,31,37,49^. Three studies reported only percentage or -fold differences between patient groups^17,30,39^. The results of these studies and those that reported on a biomarker not assessed in another study are discussed narratively. Authors of eight studies were contacted for further information^14,15,20,21,24,31,34,39^, but only those from two studies responded^15,34^.

### Risk of bias and study quality assessment

Of the included studies, twenty-three were classified as Tier 1 studies according to BIOCROSS grading and the rest were Tier 2 studies. The range of total scores varied from 10 to 17. Most of the variation was accounted for by poor scoring in BIOCROSS items 3, 4, 8, 9, 10 which indicates problems in representativeness of study populations, poor reporting of study population characteristics, inadequate details of specimen characteristics and assay methods, laboratory measurements (i.e., quality control measures, testing samples in duplicate), and insufficient information on biomarker data modelling (i.e., normal or non-normal distribution of data, handling of outliers). The scores for each BIOCROSS item for all studies are given in Supplementary Table S4.

### Biomarkers predicting the risk of dengue haemorrhagic fever (WHO 1997 classification)

**Figure 2:**
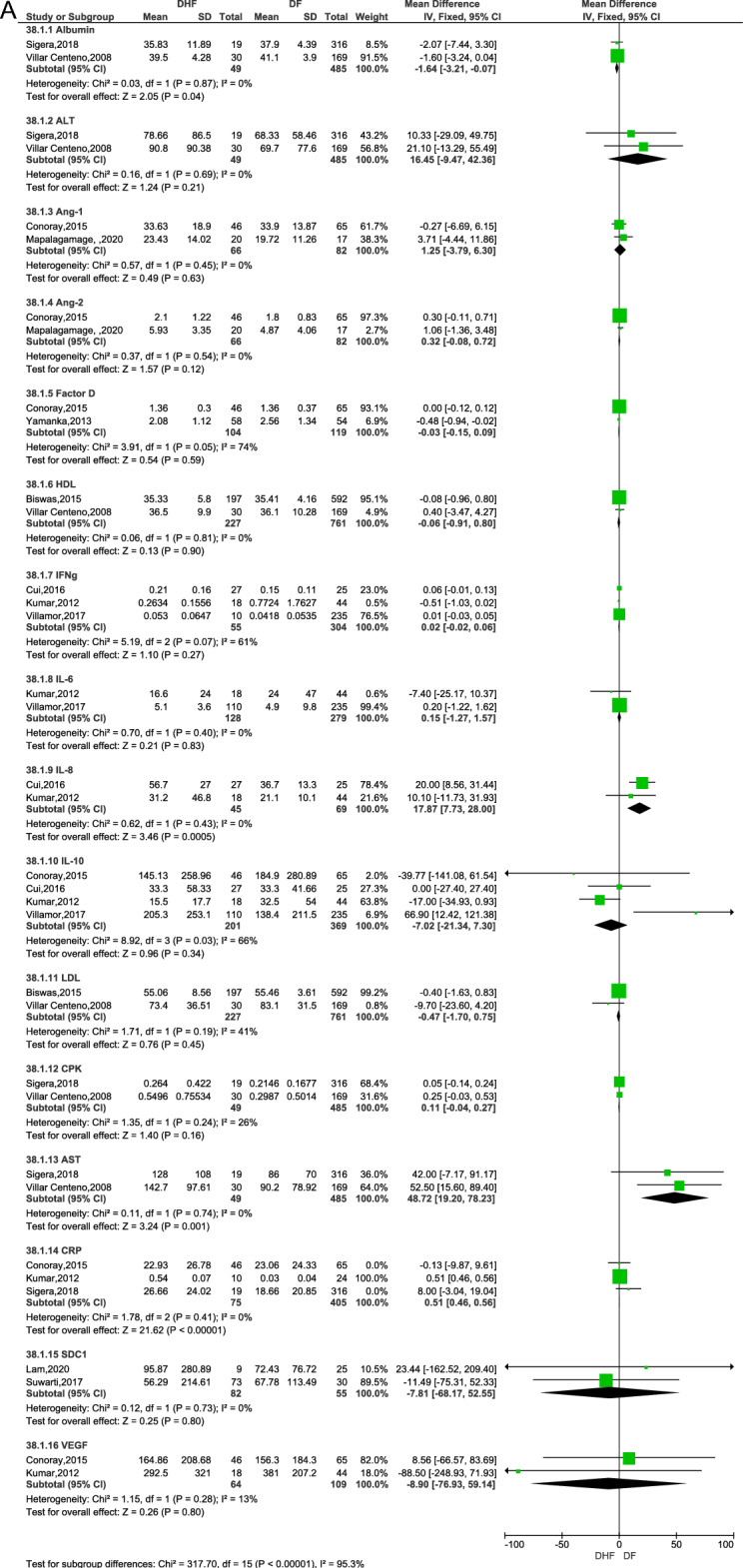

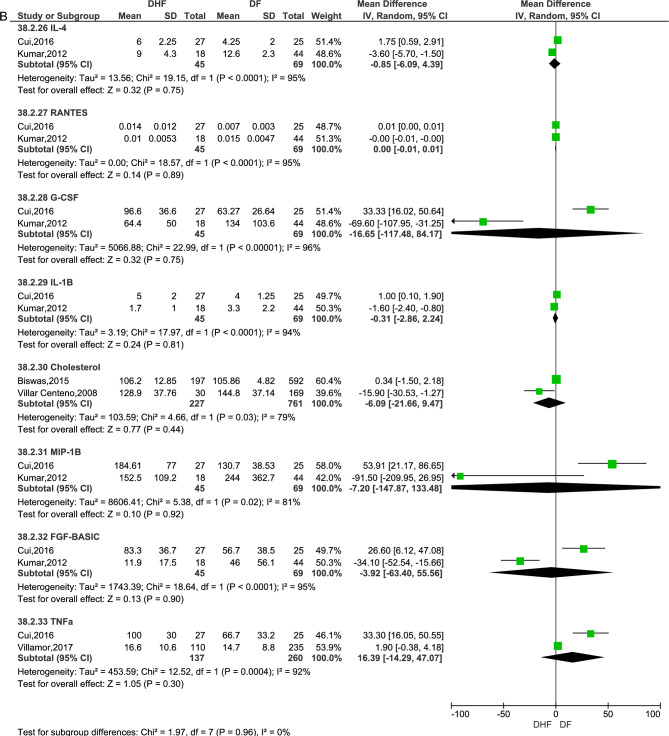
Meta-analysis of biomarkers between patients with dengue haemorrhagic fever (DHF) and those with non-DHF dengue fever (DF)—(**A**) Fixed effect model, (**B**) random effect model.

Twenty-four biomarkers assessed by eleven studies were eligible to be combined in a meta-analysis^13,15,16,22,23,28,34,36,38,40,45^ (Fig. 2a and b). Of these only four biomarkers showed statistically significant results associated with DHF. These were increased aspartate aminotransferase levels (AST, RR: 48.72, 95% CI 19.20, 78.23, 2 studies, 534 participants, p = 0.001)^34,40^, increased C-reactive protein levels (CRP, RR: 0.51, 95% CI 0.46, 0.56, 3 studies, 480 participants, p < 0.00001)^15,22,34^, increased interleukin-8 (IL-8 or CXCL8, RR: 17.87, 95% CI 7.73, 28, 2 studies, 114 participants, p < 0.0005)^16,22^, and decreased serum albumin levels (RR: − 1.64, 95% CI − 3.21, − 0.07, 2 studies, 534 participants, p = 0.04)^34,40^. The biomarkers which were not significantly associated with the risk of DHF (p > 0.05) were angiopoietin-2 (Ang-2, 2 studies, 148 participants)^15,28^, granulocyte colony stimulating factor (G-CSF, 2 studies, 114 participants) ^16,22^, interferon gamma (IFN-γ, 3 studies, 359 participants)^16,22,38^, interleukin-4 (IL-4, 2 studies, 114 participants)^16,22^, interleukin-6 (IL-6, 2 studies, 407 participants)^22,38^, interleukin-10 (IL-10, 4 studies, 570 participants) ^15,16,22,38^, interleukin 1 beta (IL-1β, 2 studies, 114 participants)^16,22^, macrophage inflammatory protein-1 beta (MIP-1β or CCL3, 2 studies, 114 participants)^16,22^, tumour necrosis factor alpha (TNF-α, 2 studies, 397participants)^16,38^, vascular endothelial growth factor (VEGF, 2 studies, 234 participants)^15,22^, Regulated upon activation normal T Cell expressed and presumably secreted chemokine (RANTES or CCL5, 2 studies, 114 participants)^16,22^, low-density lipoprotein (LDL, 2 studies, 988 participants) ^13,40^, high-density lipoprotein (HDL, 2 studies, 988 participants)^13,40^, total cholesterol (TC, 2 studies, 988 participants)^13,40^, fibroblast growth factor-basic (FGF, 2 studies, 114 participants)^16,22^, Angiopoietin-1 (Ang-1, 2 studies, 148 participants)^15,28^, complement Factor D (2 studies, 223 participants)^15,45^, alanine aminotransferase (ALT, 2 studies, 534 participants)^34,40^, creatinine phosphokinase (CPK, 2 studies, 534 participants)^34,40^, Syndecan-1 (SDC1, 2 studies, 137 participants)^23,36^. Of these G-CSF, MIP-1β and TNF-α had statistically significant differences when assessed with a fixed effect model but were noted to have high heterogeneity. When re-analysed with random effects model, the results were not statistically significant.

Fourteen studies that assessed DHF as an outcome reported on 139 biomarkers that were assessed in a single study only (no two studies assessed the same biomarker)^15–17,19,22–24,32,36,38–40,43,45^. Of these, six studies did not report variations of measurements, units of biomarker analysis or absolute abundance^14,17,20,21,24,39^. The number of participants in these 14 studies ranged from 34 to 344. Forty-four additional biomarkers were identified as being significantly (p < 0.05) associated with DHF but as mentioned before the evidence is restricted to a single study per biomarker. A full list of these biomarkers is shown in Table 3.

**Table 3:** Biomarkers statistically significantly associated with dengue haemorrhagic fever (WHO 1997 classification) but only reported in a single study.

Study	Biomarker(s)	No. of participants
Cui, 2016^16^	Serotonin, kynurenine , phenylalanyl-tryptophan, oleamide, leucyl-phenylalanine, docosenamide, deoxyinosine, leucyl-Alanine, phenylalanylphenylalanine, 3-carboxy-4-methyl-5-propyl-2-furanpropanoic acid (CMPF), N-heptanoylglycine, bilirubin, palmitic amide	52
Han, 2019^17^	Fibrinogen-like protein 1 (FGL1), microfibril-associated glycoprotein 4 (MFAP4), glutamate-ammonia ligase (GLUL), *Coagulation Factor IX (F9),* laminin subunit beta 2 (LAMB2), tissue type plasminogen activator (PLAT)	36
Kumar, 2012^22^	Serum amyloid A (SAA), histidine-phosphotransfer (HPT), platelet-derived growth factor-BB (PDGF-BB)	34
Suwarto, 2017^36^	Chondroitin sulfate, claudin-5	103
Villamor, 2018^39^	Pentadecanoic acid, stearic acid, behenic acid, 1n-7 cis-vaccenic acid, docosapentaenoic acid (DPA), docosahexaenoic acid (*DHA*), 3n-6 dihomo-γ-linolenic acid, 4n-6 arachidonic acid, 4n-6 adrenic acid, stearoyl-coA-desaturase 18, Δ5-desaturase 20	344
Villar-Centeno, 2008^40^	Lactate dehydrogenase (LDH), triglyceride (TG)	199
Houghton, 2010^19^	Soluble suppression of tumorigenesis-2 (sST2)	38
Laur, 1998^24^	Transforming growth factor beta* (*TGF*-*β*)*	52
Yamanaka, 2013^45^	Complement component 2 protein (C2), complement C4-A	112
Chaiyaratana, 2008^14^	Ferritin	117
Conroy, 2015^15^	endoglin (ENG), soluble intercellular adhesion molecules (sICAMs)	111

### Biomarkers predicting the risk of severe dengue (WHO 2009 classification)

**Figure 3:**
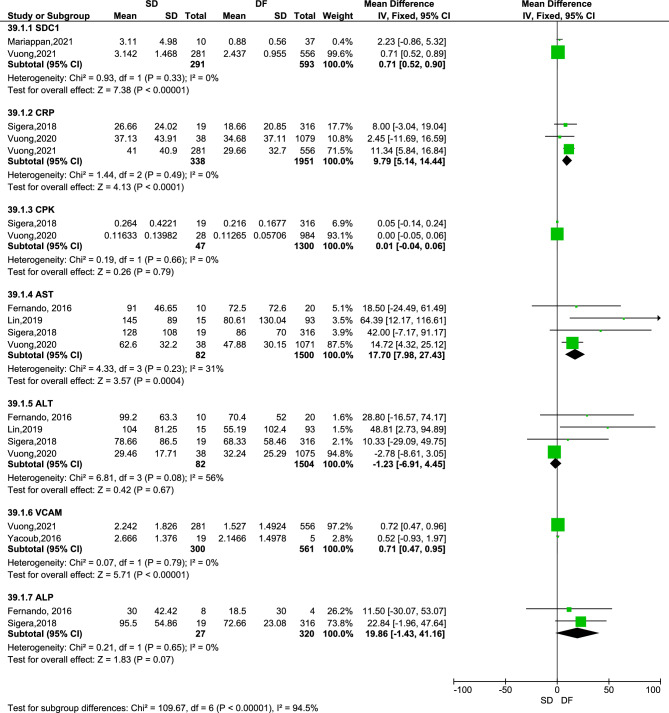
Meta-analysis of biomarkers between patients with severe dengue (SD) and others with dengue fever (DF)—fixed effect model.

Seven biomarkers assessed by seven studies were eligible to be combined in a meta-analysis^26,29,34,41,42,44,48^ (Fig. 3). Of these four biomarkers were statistically significantly associated with the risk of severe dengue. There were: increased CRP (RR: 9.79, 95% CI 5.14–14.44, 3 studies, 2289 participants, p < 0.00001)^34,41,42^, increased vascular cell adhesion protein 1 (VCAM1, RR: 709.18, 95% CI 465.43–952.93, 2 studies, 861 participants, p < 0.00001)^41,44^, increased Syndecan-1 (RR: 0.71, 95% CI 0.52–0.90, 2 studies, 884 participants, p < 0.00001)^29,41^, and increased levels of AST (RR: 17.70, 95% CI 7.98–27.43, 4 studies, 1582 participants, p = 0.0004)^26,34,42,48^. The remaining biomarkers: ALT (4 studies, 1586 participants)^26,34,42,48^, alkaline phosphatase (ALP, 2 studies, 347 participants)^34,48^, and CPK (2 studies and 1347 participants)^34,42^ were not associated with a higher risk of severe dengue (p > 0.05). Notably serum SDC1 levels were not associated with DHF, although they were significantly associated with severe dengue.

Eleven studies that assessed SD as an outcome reported on 41 biomarkers that were assessed in one study only^18,27,29–31,34,35,41,46,48,49^. Three of these studies did not report variations of measurements or units of biomarker analysis^30,31,49^. The number of participants in these 11 studies ranged from 42 to 837. Twenty-eight additional biomarkers were identified as been significantly (p < 0.05) associated with SD where the evidence is restricted to one study per biomarker. A full list of these biomarkers is shown in Table 4.

**Table 4:** Biomarkers statistically significantly associated with severe dengue (WHO 2009 classification), but only reported in a single study.

Study	Biomarker(s)	No. of participants
Mapalagamage, 2018^27^	Nitric oxide, nitrite	94
Hapugaswatta, 2021^18^	Nitric oxide synthase, oxLDL, salivary oxLDL	127
Vuong, 2021^41^	Soluble CD163, triggering receptor expressed on myeloid cells 1 (TREM1)	837
Zain, 2017^46^	Fas-ligand	42
Pang, 2016^31^	Plasminogen activator urokinase receptor (PLAUR)	80
Nhi, 2016^30^	Angiotensinogen, angiotensinogen, RIMS-binding protein 3A, WD repeat domain phosphoinositide-interacting protein, titin, ceruloplasmin, serotransferrin, zinc finger FYVE domain-containing protein 26, prothrombin nesprin-2, nesprin-2, serine palmitoyltransferase 3, nuclear pore membrane glycoprotein 210, olfactory receptor 51G2, otopetrin-3, transforming growth factor-beta-induced protein ig-h3, arf-GAP with Rho-GAP domain ANK repeat and PH domain-containing protein 3, vacuolar protein sorting-associated protein 13D, alpha-2-HS-glycoprotein, antithrombin III	50
Silva, 2021^35^	Urinary leukotriene E4 (LTE4)	120

### Sensitivity analysis

**Figure 4:**
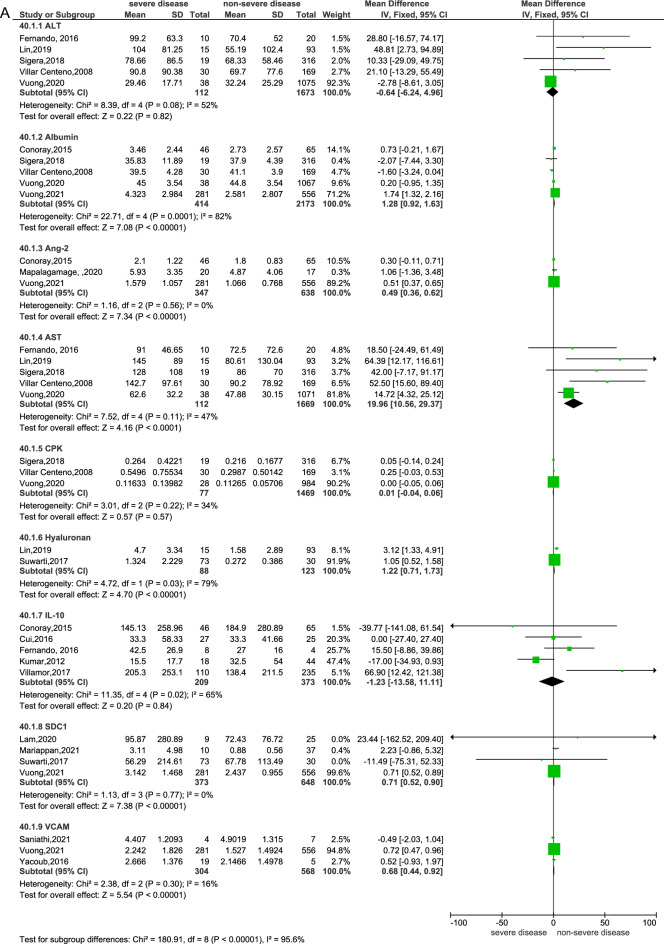

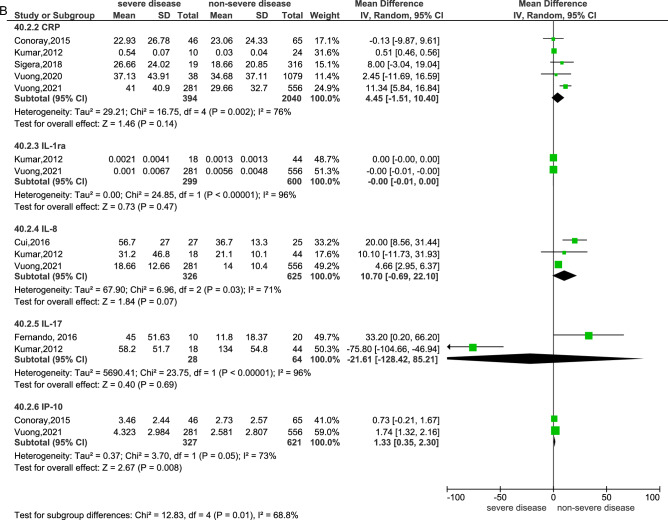
Meta-analysis of biomarkers between patients with severe disease (SD + DHF combined) and non-severe disease—(**A**) Fixed effect model, (**B**) random effect model.

When the diagnoses of both DHF and SD were combined as “severe disease”, fourteen biomarkers assessed in seventeen studies were eligible to be combined in a meta-analysis^15,16,22,23,25,26,28,29,33,34,36,39–42,44,48^ (Fig. 4a and b). Of these, six biomarkers were statistically significantly associated with the progression to severe disease. There were increased Ang-2 (RR: 0.49, 95% CI 0.36–0.62, 3 studies, 985 participants, p < 0.00001)^15,28,41^, increased AST (RR: 19.96, 95% CI 10.56–29.37), 5 studies, 1781 participants, p < 0.0001)^26,34,40,42,48^, increased SDC-1 (RR: 0.71, 95% CI 0.52–0.90, 4 studies, 1021 participants, p < 0.00001)^23,29,36,41^, increased VCAM-1 (RR: 680.15, 95% CI 438.95–921.35, 3 studies, 863 participants, p < 0.00001)^25,33,41,44^, increased interferon-gamma-induced protein-10 ( IP-10 or CXCL10, RR: 1.33, 95% CI 0.35–2.30, 2 studies, 948 participants, p = 0.008)^15,41^, and increased hyaluronan (RR: 1.22, 95% CI 0.71–1.73, 2 studies, 211 participants, p < 0.00001)^26,36^. The remaining biomarkers that did not show significant differences (p > 0.05) between severe and non-severe disease were albumin (3 studies, 1639 participants)^34,40,42^, ALT (5 studies, 1785 participants)^26,34,40,42,48^, CPK (3 studies, 1546 participants)^34,40,42^, CRP (5 studies, 2434 participants)^15,22,34,41,42^, IL-10 (5 studies, 582 participants)^15,16,22,38,48^, IL-8 (3 studies, 951 participants)^16,22,41^, interleukin-17 (IL-17, 2 studies, 92 participants)^22,48^ and interleukin-1 receptor antagonist (IL-1ra, 2 studies, 899 participants)^22,41^. Of these, CRP, IL-17, IL-1ra and IL-8 showed statistically significant results with high heterogeneity when using a fixed effects model, and the results were insignificant when re-analysed with a random effects model.

There were no data available to conduct a sensitivity meta-analysis by day of fever for any of the biomarkers. When Tier 2 studies were removed from the meta-analyses (to see if same associations remain when only studies with a lower risk of bias were considered) statistically significant results remained for all four biomarkers for DHF (albumin, CRP, AST, IL-8). However, for severe dengue (WHO 2009 criteria) only the association with AST remained (out of four previously significant biomarker associations). Similarly, for the “severe disease” category outlined in the previous paragraph, only two (CRP, AST) out of six significant associations remained after removal of Tier 2 studies. (Supplementary Fig. S1a and b).

### Ranking of biomarkers according to quality of evidence

A list of all biomarkers assessed in one or more of included studies in this review is listed accompanied with a ranking score in Supplementary File S2 according to the quality of evidence for each biomarker.

## Discussion

### Summary of main results

In this meta-analysis elevated serum CRP and AST levels in early disease (up to 96 h of fever) were associated with an increased risk of progression to either DHF (WHO 1997 classification) or severe dengue (WHO 2009 classification). In addition, early increased levels of VCAM-1 and SDC-1 were also associated with increased risk of severe dengue, while increased IL-8 and lowered albumin were associated with an increased risk of DHF. Furthermore, 44 and 28 biomarkers were identified to be statistically significantly different in those developing DHF and SD respectively, in one-off studies that needs confirming in a second study. The association with severe disease and elevated AST was consistently observed across all disease severity classifications and sensitivity analyses performed within this systematic review.

### Overall completeness and applicability of evidence

The methods of studies included in this review were highly heterogenous with different assays used to measure the same biomarker, patients from different countries and age groups included, measurements taken on different days within the 96-h period, and the final results being reported against two different dengue clinical classification systems. It is not possible to control for all these confounders without compromising the capacity to perform meta-analyses, but the quality of evidence was preserved by only including data from prospective cohort studies which conducted biomarker measurements within the first 96 h of fever, prior to the establishment of adverse disease manifestations. The results were reported separately for DHF (WHO 1997 classification) and severe dengue (WHO 2009 classification) to further reduce heterogeneity as these two clinical classifications are quite different. While there is a precedent for combining DHF and SD manifestations as “severe disease”^3^, the clinical utility of this approach is questionable as in our opinion as local guidelines in a country generally utilise one classification or the other. Overall, the findings of this review for each biomarker should only be applied within the participant (ethnicity, age group) and disease severity outcome groups for which evidence exists and should not be extrapolated to groups that were not assessed in primary research studies.

The top biomarkers identified in this review as associated with DHF have a common “hepato-centric” theme. AST elevation reflects hepatocellular injury resulting from direct viral infection or from the associated inflammation are the likely source for this observation^52–54^). CRP is an acute phase reactant produced by the liver in response to acute inflammation^55^. Serum albumin, a multi-functional plasma protein produced by the liver is a negative acute phase reactant, meaning it is downregulated in inflammatory states^56^, or it may be catabolised, or lost from the circulation in the early stages of plasma leakage. All these patterns suggest that those who go on to have DHF have a more profound early liver involvement. Interestingly, although IL-8 or neutrophil chemotactic factor was the fourth biomarker associated with DHF, neutrophil accumulation in the liver is not a feature of dengue hepatitis. This chemokine which is produced by macrophages, epithelial cells and endothelial cells not only underpins acute neutrophilic inflammation but also promotes angiogenesis^57^. Nevertheless in early dengue fever, a neutrophilia is sometimes observed, prior to the relative lymphocytosis, and IL-8 may play a role in this manifestation^58^. Again, this effect may be more profound in those who go on to develop DHF.

In relation to severe dengue, in addition to CRP and AST, SDC-1 and VCAM-1 were also significant associations. Syndecans are transmembrane domain proteins (proteoglycans) that interact with several growth factors (VEGF, FGF, TGF-β) leading to their activation, and play an important role in cell-to-cell or cell-to-extracellular matrix adhesion. Syndecan-1 is predominantly expressed in epithelial and plasma cells^59^. VCAM-1 is another cell adhesion molecule of the immunoglobulin superfamily, which is expressed on endothelial cells in inflammation after stimulation by cytokines such as TNF-α, IL-1, IL-4)^60^. Elevation of both these biomarkers selectively in those developing severe dengue is interesting and potentially hints at a direct and early role of these molecules in the disease pathogenesis and plasma leakage. Both these biomarkers may still be relevant in DHF, except that there was insufficient evidence to assess in the meta-analysis. Given that patients with severe dengue generally have worse outcomes than DHF patients^4^, it is plausible that early biomarker profiles associated with these two outcomes may be different. By contrast, the meta-analyses reveal that CRP and AST are both early biomarkers of severe manifestations regardless of the outcome classification. It is interesting that elevation of ALT, the second transaminase which is also released with hepatocyte necrosis and hepatic inflammation was not associated with either SD or DHF. This raises the possibility that part of the AST elevation in serum may come from skeletal muscle, given that myalgia is a prominent symptom in early dengue infection.

### Quality of the evidence

The biomarkers were arranged according to the quality of evidence in Supplementary File S2 in the following categories. Given the absence of a precedence to classify evidence in a similar review, these criteria were developed by the authors specifically for this review. The quality of evidence is highest for the categories of either extreme (categories I and VIII) and declines towards the middle.

- Category I—confirmed as statistically significant in a meta-analysis.
- Category II—confirmed as statistically significant in two or more studies that cannot be combined in a meta-analysis due to inadequate data reporting, differences in units of measurement or other reasons.
- Category III—confirmed as statistically significant in one Tier 1 study.
- Category IV—confirmed as statistically significant in one Tier 2 study.
- Category V—confirmed as statistically insignificant in one Tier 2 study.
- Category VI—confirmed as statistically insignificant in one Tier 1 study.
- Category VII—confirmed as statistically insignificant in two or more studies that cannot be combined in a meta-analysis.
- Category VIII—confirmed as statistically insignificant in a meta-analysis.

### Potential biases of the review process

As mentioned, the evidence presented in this systematic review comes mostly from studies in South or South-eastern Asian and Latin American countries. There may be ethnic (including genetic and environmental factors), as well as viral serotype determined differences in immunity to dengue virus, which limits the extrapolation of results beyond the communities that were assessed in the primary research. Importantly this review does not differentiate between adults and children. As the criterion for inclusion children was different in each country (ranging from 12 to 18 years), it was not possible to have a uniform age consensus to extract data for a separate meta-analysis in children. It was also not possible to differentiate between primary and secondary dengue or infecting serotype as only a few eligible studies reported results stratified by these variables. It is well established that due to non-neutralising cross-reactive antibodies, secondary dengue infections from a different serotype are more likely to have severe manifestations. Finally, this analysis considers the first 96 h of infection as a “homogenous” period in the course of illness. This is not likely to be accurate as the disease evolves considerably even within this short time window. While an attempt was made to separate results by day of fever in the sensitivity analysis, it was not possible to do a meta-analysis for any of the biomarkers, due to inconsistent and inadequate reporting across eligible studies.

### Agreements and disagreements with other similar reviews

The most recent similar systematic review by Thach et al.^10^ on this topic had a search date of 20th September 2020, 1.5 years prior to the search date of this review. Six included studies in the current systematic review were published in 2021, and hence were not considered in the previous review. Thach et al. only considered data within the first 72 h of fever, while this review extended the period of interest to 96 h, provided an adverse outcome had not been recorded within this time window or at admission. The current review was restricted to studies with biomarkers, providing an in-depth exhaustive analysis of various biomarkers assessed for associations with severe disease paying attention to both significant and insignificant associations. The previous review also considered cell or platelet counts, viral determinants (e.g., viral load), as well as clinical symptoms and signs, and hence the focus and data generated on biomarkers was considerably less with results presented only for biomarkers that were combined in a meta-analysis. The risk of bias in the previous review was assessed using the National Heart, Lung, and Blood Institute (NHLBI) tool^61^, whereas the BIOCROSS tool^50^ used in this review includes all relevant aspects covered by the NHBLI tool, but also has additional domains more tailored to quality assessment of biomarker studies. It is a challenge to find a tool to evaluate the quality of studies that have assessed biomarkers as most tools focus on clinical study designs and patient focussed outcomes (e.g., NHLBI tools). Problems such as reliability of assay measurements, test to test variation are not addressed in these tools. In our opinion, BIOCROSS is a more detailed, comprehensive tool which considers the errors in biomarker measurement and hence more suited for this type of review. The outcome classifications in the previous review are also different with DHF grades III and IV (WHO 1997 classification) and severe dengue (WHO 2009 classification) combined as “severe disease” and everything else as “non-severe”. The current review avoided combining different classification systems in the main analysis for ease of interpretation in patient management and better utilisation of data in research as most management guidelines and research studies use one classification or the other (rather than a combination). Despite these differences, both reviews agree that serum AST is a significant association for severe disease, and that serum ALT is not. Thach et al. did not observe a significant association with CRP and albumin levels with severe disease. although these results should be interpreted in the context of differences highlighted above. Data on IL-8, SDC-1 or VCAM-1 were not mentioned in the previous review.

Two other recent reviews by Sangkaew et al.^3^ (last date of search: 31st January 2020) and Yuan et al.^9^ (last date of search: December 2020) also assessed predictors for severe disease manifestations. However, both reviews considered a large number of demographic, clinical, immunological and virological parameters to process, and so considered biomarkers in limited detail. For example, Sangkaew et al. only reported variables that had been reported in four or more studies only, which reduced their reporting to only three biomarkers (elevated AST, ALT, and reduced albumin levels) presumably because these were the only statistically significant associations for severe disease. Sangkaew et al. combined clinical classifications from the WHO 1997 and 2009 guidelines to define a new “severe disease” category for the purpose of meta-analysis. For reasons mentioned above, the current review kept these classifications separate in the main analysis, but in sensitivity analysis the approach taken by Sangkaew et al. was followed. In that sensitivity analysis our results were similar for AST, but not for ALT or albumin, but these differences could not be further examined as individual studies backing these observations could not be identified from the previous review. It is noted however that when multiple observations were made of a biomarker during the febrile phase, Sangkaew et al. only considered the value reported on day 3 of fever while the current review used the average of all readings. The other review by Yuan et al. reported on several biomarkers, but the inclusion criteria were not restricted to the data from the first 72–96 h of fever or the “febrile” phase. Hence, the clinical utility of this review is limited in identifying early predictive biomarkers for severe disease and therefore, its findings cannot be compared with the present analysis.

## Conclusions

### Implications for practice

This review identified several biomarkers that are differentially expressed in early disease in those who subsequently develop SD or DHF. Markers such as CRP, AST, and albumin are routinely measured even in resource limited settings as part of disease management. Clinicians and guideline development committees should consider analysing existing datasets to identify appropriate cut-off values for these parameters which can then be used in a clinical risk prediction system in combination with other non-biomarker severity predictors.

### Implications for research

Biomarkers IL-8, VCAM-1 and Syndecan-1 identified in this review as predictors do not have low-cost tests to assess their concentration in human serum. Development of such tests will further enhance the predictive value of risk scoring systems within resource limited settings. In addition, there are many biomarkers already shown to be associated with severe disease manifestations in a single study where confirmation by a second study would increase the quality and certainty of evidence through a meta-analysis (biomarkers in categories II – IV in the quality of evidence ranking system). Prioritising immediate research on these biomarkers would be cost effective and potentially high yield in terms of generating clinically useful evidence for predicting severe manifestations of dengue fever.

### Differences between review and protocol

The title of the protocol “early prognostic biomarkers for severe dengue and plasma leakage” was changed as the review encompasses all adverse manifestations namely severe dengue, DHF and plasma leakage. The search strategy was altered to capture relevant more articles than initially intended. The exclusion criteria were expanded as indicated above in the review compared to the protocol. DHF was included as a comparator group to the review (not mentioned in protocol) given the large number of studies using the 1997 WHO clinical classification for dengue.

### Supplementary Information

Supplementary Information 1.

Supplementary Information 2.

## Data Availability

All data generated or analysed during this study are included in this published article (and its Supplementary Information files).
