# Supplementary material for: Early biomarkers for prediction of severe manifestations of dengue fever: a systematic review and a meta-analysis
Source: Sci Rep. 2023 Oct 14;13:17485. doi: 10.1038/s41598-023-44559-9 (PMC10576797; doi:10.1038/s41598-023-44559-9)
Supplement: Supplementary file 2 — Supplementary Information 2. [file 41598_2023_44559_MOESM2_ESM.pdf]

Supplementary material

Supplementary Tables

STS1. Exclusion criteria of publications

STS2. Characteristics of excluded studies

STS3. BIOCROSS quality assessment tool with minor modifications

STS4. BIOCROSS grading of each study

Supplementary figures

Supplementary figure S1. Repeat meta-analysis after removing tier 2 studies – 1A: severe dengue (SD) vs. others, 1B: severe disease (SD + DHF) vs. others.

Supplementary file S1: Minimum dataset of the analysis

Supplementary file S2: Ranking of biomarkers according to certainty of evidence.

Supplementary file S3: PRISMA Checklist for systematic reviews

Supplementary Table S1. Exclusion criteria of publications

**List of exclusion criteria**
Articles published before 1997
In vitro or animal studies
Study designs other than prospective cohorts or nested case control studies (e.g., retrospective studies, case-control studies)
Studies reporting secondary data (systematic, narrative and scoping reviews)
Other papers describing the same cohort for which the data had been extracted, unless reporting on a new biomarker
Studies not reporting biomarker measurements within the first 96 hours of fever
Studies without laboratory confirmation of dengue fever
Studies not reporting comparisons of appropriately measured biomarkers between at least ONE of the following patient groups:
• With plasma leakage vs. without plasma leakage
• With DHF vs. without DHF (1997 WHO classification)
• With severe dengue vs. without severe dengue (2009 WHO classification)
Studies that only report on following as risk factors:
• Genetic polymorphisms
• mRNA / transcriptomics
• RBC, WBC, platelet cell counts
• Clinical signs and symptoms
• Virological determinants (serotype, viral load)
• Demographic determinants

Supplementary Table S2. characteristics of excluded studies

Study	Reason
Cui,2013	Compares DF* vs. healthy controls, but not with DHF*
Green,1999	Compares the outcomes based on the day of defervescence.
Hadinegoro, 1999	Compares different grades of DHF* without a DF* category
Iani,2016	Comparisons are based on the presence or absence of haemorrhagic manifestations only
Jeewandara,2015	Biomarker data not provided
Mairuhu,2005	Compares DF* and DHF* (together) vs. DSS*
Neguyen,2013	WHO* clinical classification of dengue is not reported
Potts,2010	The biomarker assay is not described. No data is available for the measured biomarker.
Shyamali,2020	Data for early days of fever cannot be differentiated
Soundravally, ,2008	Some patients had DHF* at the time of presentation
Trung,2010	Comparisons are based on the presence or absence of bleeding only
Van Ta, 2019	Only compares DHF* I and II vs. III and IV, no DF* category

Villar-Centeno,2013	Biomarker data was not provided, ROC curve and cut-off points were reported

*Abbreviations: DF, dengue fever; DHF, dengue haemorrhagic fever, DSS – Dengue shock syndrome, WHO – World Health Organization

Supplementary Table S3. BIOCROSS[1] quality assessment tool with minor modifications

**BIOCROSS evaluation tool**.
**Domain 1: Study rationale**
**Item 1 (Hypothesis/Objectives)**
1. 1.1 Was the biomarker under study described?
1.2 Was the rationale for the study (research question) clearly presented?
1.3 Were the study objectives/ hypothesis clearly stated?
**Domain 2: Design / Methods**
**Item 2 (study population)**
2. 2.1 Were the characteristics (day of sampling- Plasma leakage /non-Plasma leakage) of the study participants presented?
2.2 Were the disease stages (DF/DHF/DSS) of the included participants described?
2.3 Were the inclusion and exclusion criteria for study participation defined?
**Item3 (study population representatives)**
3. 3.1 Was the sampling frame reported (study population source)
3.2 Was the participation rate reported (i.e. eligible persons at least 50%)?
3.3 Was sample size justification or power description provided?

**Domain 3: Data analysis**
**Item 4 (Study population characteristics)**
4. 4.1 Were the study population characteristics (i.e. demographic, clinical and social) presented?
4.2 Were the exposures and diagnostic methods described?
4.3 Were any missing values and strategies to deal with missing data reported?

**Item 5 (Statistical analysis)**
5. 5.1 Did the authors clearly report statistical methods used to calculate estimates (e.g. Spearman/Pearson/Linear regression, etc.)?
5.2 Were key potential confounding variables measured and adjusted statistically in reported analyses?
5.3 Was the raw effect size estimate (correlation coefficient, beta coefficient) or measure of study
precision provided (e.g. confidence intervals, precise (!) p-value*)?
**Domain 4: Data interpretation**
**Item 6 (Interpretation and evaluation of results)**
6. 6.1 Was the data discussed in the context of study objectives/hypotheses?
6.2 Was the interpretation of the results considering findings from similar studies?
6.3 Was the biological context described?
**Item 7 (Study limitations)**
7. 7.1 Was the prospective cohort or nested-case control nature of the analysis discussed?
7.2 Did the authors acknowledge restricted interpretation due to measurements at one point in time and no statement about causality possible using prospective studies?
7.3 Did the authors acknowledge the need for consistency with other research?
**Domain 5: Biomarker measurement**
**Item 8 (Specimen characteristics and assay methods)**
8. 8.1 Were the measurement methods described? (assay methods, preservation and storage, detailed protocol, including specific reagents or kits used)
8.2 Were the reproducibility assessments performed for evaluating biomarker stability?
8.3 Were the quantitation methods well described?
**Item 9 (Laboratory measurement)**
9. 9.1 Was the laboratory/place of measurement mentioned?
9.2 Were any quality control procedures and results reported (e.g. reported coefficient of variation?

9.3 Were the analyses blinded for laboratory staff?
**Item 10 (Biomarker data modelling)**
10.1 Was the distribution of biomarker data reported (if non-normal how it was standardized)?
10.2 Did the authors report on methods or outlier detection and handling?
10.3 Were any possible errors resulting from measurement inaccuracies discussed?

BIOCROSS is a 10-item tool covering 5 domains. each item represents quality features assessed in each domain and consists of 3 issues to consider (ICs). The evaluation score for each IC is ordinal scale (0,1,2) which allows the tool to have a gradual rating. When all 3 ICs in each item are well discussed, a score of 2 should be given to the item, if 1 or 2 IC were discussed in a feasible way, a score of 1 and if no IC was well explained, 0 point should be awarded. This scoring results in a maximum of 20 scores for each study.

Supplementary Table S4. BIOCROSS grading of each study (to be read in conjunction with Supplementary table 3)

Study	Score per each item in the BIOCROSS tool										Total
	1	2	3	4	5	6	7	8	9	10	
Biswas,2015	2	2	1	1	1	2	2	0	1	0	12
Chaiyaratana, 2008	2	2	1	1	1	1	1	1	0	0	10
Conroy, 2015	2	2	1	1	2	2	2	2	1	1	16*
Cui, 2016	2	2	1	1	2	2	2	2	1	1	16*
Han, 2019	2	2	1	1	2	2	2	1	1	1	15*
Hapugaswatta,2021	2	2	1	1	2	1	1	2	1	1	14
Houghton,2010	2	2	1	1	2	2	2	1	1	0	14
Koraka, 2004	2	2	1	1	2	2	2	1	1	1	15*
Kularatnam,2019	2	2	1	1	2	2	2	1	1	1	15*
Kumar, 2012	2	2	1	2	2	2	2	1	1	1	16*
Lam,2020	2	2	1	2	2	2	2	1	2	0	16*
Laur, 1998	2	2	1	1	1	2	2	0	0	0	11

Liao,2015	2	2	1	1	2	2	2	1	1	1	15*
Lin,2019	2	2	1	1	2	2	2	2	1	1	16*
Mapalagamage, 2018	2	2	1	1	2	2	2	1	1	1	15*
Mapalagamage, 2020	2	2	1	1	2	2	2	2	1	1	16*
Mariappan,2021	2	2	1	1	2	2	2	1	1	1	15*
Nhi,2016	2	2	1	2	2	2	2	1	1	1	16*
Pang,2016	2	2	1	2	2	2	2	1	1	1	17*
Rathore, 2020	2	2	1	1	2	1	1	1	1	1	13
Saniathi, 2021	2	2	1	0	0	2	2	0	1	1	11
Sigera, 2019	1	2	2	1	2	2	2	2	1	1	16*
Silva,2021	2	2	1	1	2	2	2	1	1	1	15*
Suwarto, 2017	2	2	1	2	2	2	2	2	1	1	17*
van de Weg, 2014	2	2	1	1	2	2	2	1	1	1	15*

Villamor, 2017	2	2	1	2	2	2	2	1	1	1	15*
Villamor,2018	2	2	1	1	2	2	2	1	1	1	16*
Villar-Centeno, 2008	2	2	1	1	2	2	2	1	1	1	15*
Vuong, 2021	2	2	1	1	1	2	2	1	1	1	14
Vuong, 2020	2	2	1	1	2	2	2	1	1	1	15*
Yacoub, 2017	2	2	1	1	2	2	2	1	1	1	15*
Yacoub, 2016	2	2	1	1	1	2	2	1	0	1	13
Yamanaka, 2013	2	2	1	1	2	2	2	1	0	1	14
Zain, 2017	2	2	1	1	1	2	2	1	0	1	13
Chandrashekhar, 2019	2	2	2	1	1	2	1	0	0	0	11
Fernando, 2016	1	2	1	1	1	2	1	1	0	0	10
Low, 2018	2	2	2	1	1	2	1	1	1	0	13

*Tier 1 studies with a BIOCROSS grading > 14

1. Wirsching, J., et al., *Development and reliability assessment of a new quality appraisal tool for cross-sectional studies using biomarker data (BIOCROSS).* BMC Med Res Methodol, 2018. **18**(1): p. 122.

Study or Subgroup	SD Mean	SD SD	SD Total	DF Mean	DF SD	DF Total	Weight	Mean Difference IV, Fixed, 95% CI
**41.1.1 CRP**								
Sigera,2018	26.66	24.02	19	18.66	20.85	316	62.1%	8.00 [-3.04, 19.04]
Vuong,2020	37.13	43.91	38	34.68	37.11	1079	37.9%	2.45 [-11.69, 16.59]
**Subtotal (95% CI)**			**57**			**1395**	**100.0%**	**5.90 [-2.81, 14.60]**
Heterogeneity: Chi² = 0.37, df = 1 (P = 0.54); I² = 0%								
Test for overall effect: Z = 1.33 (P = 0.18)								
**41.1.2 ALT**								
Lin,2019	104	81.25	15	55.19	102.4	93	1.5%	48.81 [2.73, 94.89]
Sigera,2018	78.66	86.5	19	68.33	58.46	316	2.1%	10.33 [-29.09, 49.75]
Vuong,2020	29.46	17.71	38	32.24	25.29	1075	96.4%	-2.78 [-8.61, 3.05]
**Subtotal (95% CI)**			**72**			**1484**	**100.0%**	**-1.71 [-7.43, 4.01]**
Heterogeneity: Chi² = 5.10, df = 2 (P = 0.08); I² = 61%								
Test for overall effect: Z = 0.59 (P = 0.56)								
**41.1.3 AST**								
Lin,2019	145	89	15	80.61	130.04	93	3.7%	64.39 [12.17, 116.61]
Sigera,2018	128	108	19	86	70	316	4.1%	42.00 [-7.17, 91.17]
Vuong,2020	62.6	32.2	38	47.88	30.15	1071	92.2%	14.72 [4.32, 25.12]
**Subtotal (95% CI)**			**72**			**1480**	**100.0%**	**17.66 [7.68, 27.64]**
Heterogeneity: Chi² = 4.32, df = 2 (P = 0.12); I² = 54%								
Test for overall effect: Z = 3.47 (P = 0.0005)								

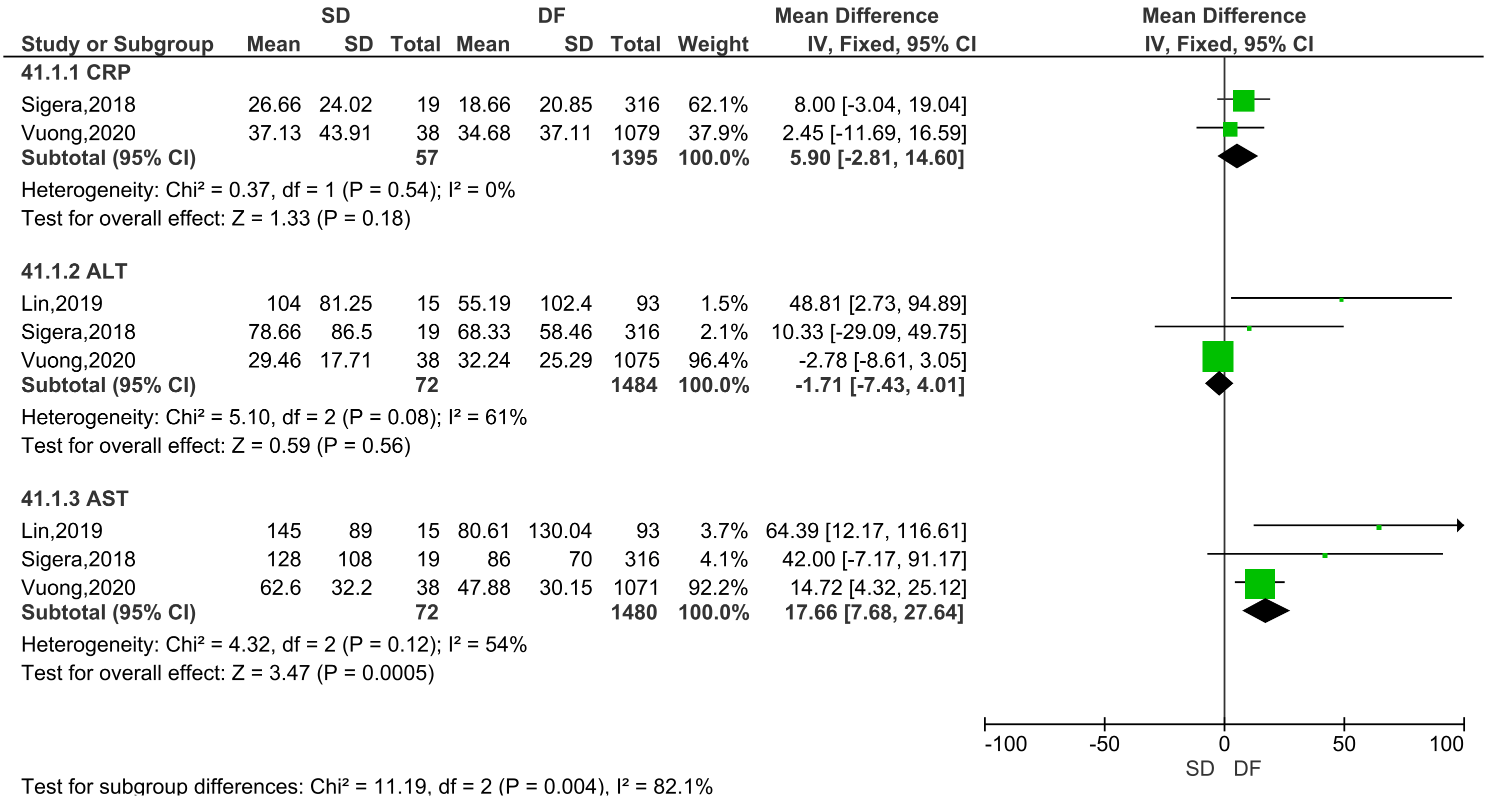

Test for subgroup differences: Chi² = 11.19, df = 2 (P = 0.004), I² = 82.1%

Supplementary figure S1A. Repeat meta-analysis after removing tier 2 studies – severe dengue (SD) vs. others

Study or Subgroup	Severe disease Mean	SD	Total	DF Mean	SD	Total	Weight	Mean Difference IV, Fixed, 95% CI
**41.2.1 CRP**								
Conoray,2015	22.93	26.78	46	23.06	24.33	65	0.0%	-0.13 [-9.87, 9.61]
Kumar,2012	0.54	0.07	10	0.03	0.04	24	100.0%	0.51 [0.46, 0.56]
Sigera,2018	26.66	24.02	19	18.66	20.85	316	0.0%	8.00 [-3.04, 19.04]
Vuong,2020	37.13	43.91	38	34.68	37.11	1079	0.0%	2.45 [-11.69, 16.59]
**Subtotal (95% CI)**			**113**			**1484**	**100.0%**	**0.51 [0.46, 0.56]**
Heterogeneity: Chi² = 1.86, df = 3 (P = 0.60); I² = 0%								
Test for overall effect: Z = 21.62 (P < 0.00001)								
**41.2.2 SDC-1**								
Lam,2020	95.87	280.89	9	72.43	76.72	25	0.0%	23.44 [-162.52, 209.40]
Mariappan,2021	3.11	4.98	10	0.88	0.56	37	99.7%	2.23 [-0.86, 5.32]
Suwarti,2017	56.29	214.61	73	67.78	113.49	30	0.2%	-11.49 [-75.31, 52.33]
**Subtotal (95% CI)**			**92**			**92**	**100.0%**	**2.20 [-0.88, 5.29]**
Heterogeneity: Chi² = 0.23, df = 2 (P = 0.89); I² = 0%								
Test for overall effect: Z = 1.40 (P = 0.16)								
**41.2.3 ALT**								
Lin,2019	104	81.25	15	55.19	102.4	93	1.5%	48.81 [2.73, 94.89]
Sigera,2018	78.66	86.5	19	68.33	58.46	316	2.1%	10.33 [-29.09, 49.75]
Villar Centeno,2008	90.8	90.38	30	69.7	77.6	169	2.7%	21.10 [-13.29, 55.49]
Vuong,2020	29.46	17.71	38	32.24	25.29	1075	93.8%	-2.78 [-8.61, 3.05]
**Subtotal (95% CI)**			**102**			**1653**	**100.0%**	**-1.09 [-6.74, 4.55]**
Heterogeneity: Chi² = 6.75, df = 3 (P = 0.08); I² = 56%								
Test for overall effect: Z = 0.38 (P = 0.70)								
**41.2.4 AST**								
Lin,2019	145	89	15	80.61	130.04	93	3.4%	64.39 [12.17, 116.61]
Sigera,2018	128	108	19	86	70	316	3.8%	42.00 [-7.17, 91.17]
Villar Centeno,2008	142.7	97.61	30	90.2	78.92	169	6.8%	52.50 [15.60, 89.40]
Vuong,2020	62.6	32.2	38	47.88	30.15	1071	85.9%	14.72 [4.32, 25.12]
**Subtotal (95% CI)**			**102**			**1649**	**100.0%**	**20.04 [10.40, 29.67]**
Heterogeneity: Chi² = 7.52, df = 3 (P = 0.06); I² = 60%								
Test for overall effect: Z = 4.07 (P < 0.0001)								
**41.2.5 Ang-2**								
Conoray,2015	2.1	1.22	46	1.8	0.83	65	97.3%	0.30 [-0.11, 0.71]
Mapalagamage, ,2020	5.93	3.35	20	4.87	4.06	17	2.7%	1.06 [-1.36, 3.48]
**Subtotal (95% CI)**			**66**			**82**	**100.0%**	**0.32 [-0.08, 0.72]**
Heterogeneity: Chi² = 0.37, df = 1 (P = 0.54); I² = 0%								
Test for overall effect: Z = 1.57 (P = 0.12)								

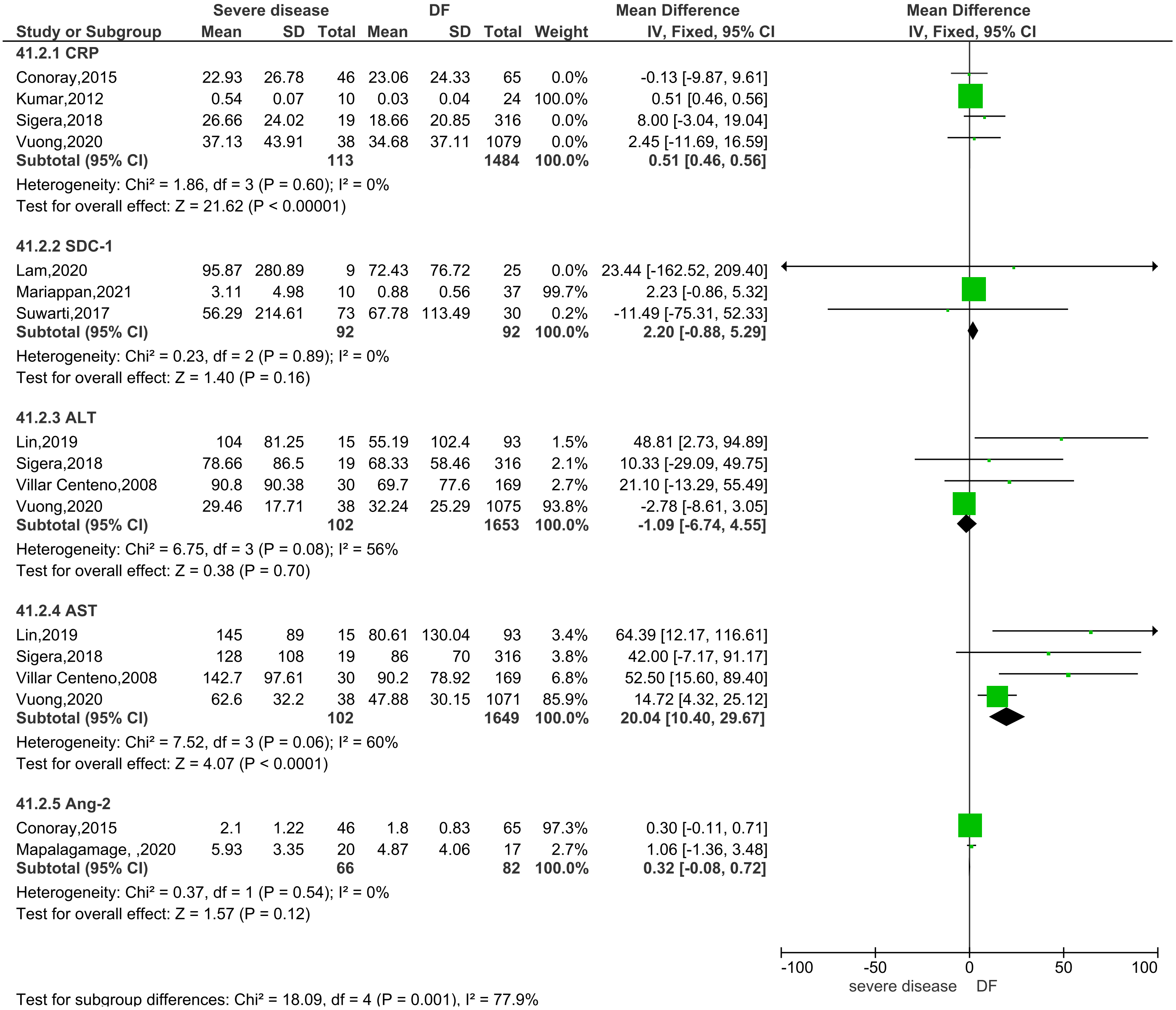

Test for subgroup differences: Chi² = 18.09, df = 4 (P = 0.001), I² = 77.9%

Supplementary figure S1B. Repeat meta-analysis after removing tier 2 studies – Severe disease (SD + DHF) vs. others.

Supplementary file S2: Ranking of biomarkers according to certainty of evidence

Biomarker(222 biomarkers)	Number of participants	Highest Biocross grade	Ranking category*	Study names
Albumin	534	16	1	Sigera,2019
Ang-2	985	16	1	Conoray,2015
AST	1781	16	1	Fernando,2016
CRP	2434	16	1	Conoray,2015
IP-10	984	16	1	Conoray,2015
SDC1	1021	17	1	Mariappan,2021
VCAM	863	14	1	Saniathi,2021
IL-8	951	16	1	Cui,2016
Hyaluronan	211	17	1	Suwarti,2017
Alpha-2-HS-glycoprotein	71	16	3	Nhi,2016
Total bilirubin	2	16	3	Sigera, 2018
C4(c4a)	112	16	3	Yamanaka,2013
sENG	111	16	3	Conoray,2015
sICAM-1	111	17	3	Conoray,2015
Serotonin	52	16	3	Cui,2016
Kynurenine	52	16	3	Cui,2016
phenylalanyl-tryptophan	52	16	3	Cui,2016
oleamide	52	16	3	Cui,2016
Leucyl-phenylalanine	52	16	3	Cui,2016
Docosenamide	52	16	3	Cui,2016
Deoxyinosine	52	16	3	Cui,2016
Leucyl-Alanine	52	16	3	Cui,2016
Palmitic amide(acid)	344	16	3	Cui,2016
PS (18:0/18:0)	52	16	3	Cui,2016
Phenylalanylphenylalanine	52	16	3	Cui,2016

CMPF	52	16	3	Cui,2016
LPE(22:4/0:0)	52	16	3	Cui,2016
PE(18:1/22:5)	52	16	3	Cui,2016
PE (20:4/20:3)	52	16	3	Cui,2016
PE(20:4/P-18:1)	52	16	3	Cui,2016
LPE(20:0/0:0)	52	16	3	Cui,2016
PS(18:0/20:0)	52	16	3	Cui,2016
N-Heptanoylglycine	52	16	3	Cui,2016
PLAT	36	15	3	Han,2019
LAMB2	36	15	3	Han,2019
F9	36	15	3	Han,2019
FGL1	36	15	3	Han,2019
MFAP4	36	15	3	Han,2019
GLUL	36	15	3	Han,2019
PDGF-BB	34	16	3	Kumar,2012
SAA	34	16	3	Kumar,2012
HPT	34	16	3	Kumar,2012
NOx (Serum)	94	15	3	Mapalagamage,2018
Nitrite (Serum)	94	15	3	Mapalagamage,2018
uPAR (PLAUR)	80	17	3	Pang,2016
Angiotensinogen	50	16	3	Nhi,2016
RIMS-binding protein 3A	50	16	3	Nhi,2016
WD repeat domain Phosphoinositide-interacting protein 2	50	16	3	Nhi,2016
Antithrombin III	50	16	3	Nhi,2016
Titin	50	16	3	Nhi,2016
Ceruloplasmin	50	16	3	Nhi,2016
Serotransferrin	50	16	3	Nhi,2016
Zinc finger FYVE domain-containing protein 26	50	16	3	Nhi,2016

Prothrombin Nesprin-2	50	16	3	Nhi,2016
Nesprin-2	50	16	3	Nhi,2016
Multidrug resistance-associated protein 6	50	16	3	Nhi,2016
Serine palmitoyltransferase 3	50	16	3	Nhi,2016
Nuclear pore membrane glycoprotein 210	50	16	3	Nhi,2016
Olfactory receptor 51G2	50	16	3	Nhi,2016
Otopetrin-3	50	16	3	Nhi,2016
Transforming growth factor-beta-induced protein ig-h3	50	16	3	Nhi,2016
Arf-GAP with Rho-GAP domain, ANK repeat and PH domain-containing protein 3	50	16	3	Nhi,2016
Vacuolar protein sorting-associated protein 13D	50	16	3	Nhi,2016
urinary LTE4	120	15	3	Silva,2021
Chondroitin sulfate	103	17	3	Suwarto,2017
Claudin-5	103	17	3	Suwarto,2017
15 : 0 pentadecanoic acid	344	16	3	Villamore,2018
18 : 0 stearic acid	344	16	3	Villamore,2018
22 : 0 behenic acid	344	16	3	Villamore,2018
Total SFA	344	16	3	Villamore,2018
18 : 1n-7 cis-vaccenic acid	344	16	3	Villamore,2018
22 : 5n-3 DPA	344	16	3	Villamore,2018
22 : 6n-3 DHA	344	16	3	Villamore,2018
Total long-chain n-3 PUFA†	344	16	3	Villamore,2018
Total n-3 PUFA	344	16	3	Villamore,2018
20 : 3n-6 dihomo-γ-linolenic acid	344	16	3	Villamore,2018
20 : 4n-6 arachidonic acid	344	16	3	Villamore,2018
22 : 4n-6 adrenic acid	344	16	3	Villamore,2018
Total long-chain n-6 PUFA‡	344	16	3	Villamore,2018
LDH	199	15	3	Villar Centeno,2008

TG	199	15	3	Villar Centeno,2008
iNOS	127	14	4	Hapugaswatta,2021
plasma oxLDL	127	14	4	Hapugaswatta,2021
Salivary oxLDL	27	14	4	Hapugaswatta,2021
sSt2	38	14	4	Houghton,2010
sCD163	837	14	4	Vuong,2021
sTREM-1	837	14	4	Vuong,2021
C2	112	14	4	Yamanaka,2013
Fasligand	42	13	4	Zain,2017
TGF-b	52	11	4	Laur,1998
IL-18	2	17	5	Conoray,2015
CHI3L1	1	16	5	Conoray,2015
C5a	1	16	5	Conoray,2015
sTie-1	1	16	5	Conoray,2015
sTie-2	1	16	5	Conoray,2015
Angptl3	1	16	5	Conoray,2015
Angptl4	1	16	5	Conoray,2015
sFlt-1	1	16	5	Conoray,2015
Skdr	1	16	5	Conoray,2015
Platelet factor 4a	1	16	5	Conoray,2015
IL-5	1	16	5	Kumar,2012
IL-7	1	16	5	Kumar,2012
IL-9	1	16	5	Kumar,2012
IL-12	1	16	5	Kumar,2012
IL-13	1	16	5	Kumar,2012
Eotaxin	1	16	5	Kumar,2012
IP-101	1	16	5	Kumar,2012
Serum Amyloid A2**	1	16	5	Kumar,2012
Leucine-rich-alpha-2 glycoprotein**	1	16	5	Kumar,2012
Hemoglobin subunit-alpha**	1	16	5	Kumar,2012

Actin, cytoplasmic**	1	16	5	Kumar,2012
Hemoglobin subunit delta	1	16	5	Kumar,2012
Insulin-like growth factor-binding protein 3	1	16	5	Kumar,2012
Hemoglobin subunit-beta	1	16	5	Kumar,2012
Phosphatidyl-inositol glycan specific	1	16	5	Kumar,2012
phospholipase-D	1	16	5	Kumar,2012
Plasma protease C1 inhibitor	1	16	5	Kumar,2012
Hemoglobin subunit zeta	1	16	5	Kumar,2012
Coagulation factor XII	1	16	5	Kumar,2012
EGF-containing fibulin-like ECM protein	1	16	5	Kumar,2012
Alpha-1B-glycoprotein	1	16	5	Kumar,2012
Tumor protein 63	1	16	5	Kumar,2012
Alpha-2 antiplasmin	1	16	5	Kumar,2012
Alpha-1-antichymotrypsin	1	16	5	Kumar,2012
Hemopexin	1	16	5	Kumar,2012
Transthyretin	1	16	5	Kumar,2012
Complement component C9	1	16	5	Kumar,2012
Inter-alpha-trypsin inhibitor H3	1	16	5	Kumar,2012
Alpha-1-acid glycoprotein 1	1	16	5	Kumar,2012
Haptoglobin**	1	16	5	Kumar,2012
Inter-alpha-trypsin inhibitor H2	1	16	5	Kumar,2012
Apolipoprotein A-I	1	16	5	Kumar,2012
Serum Paraoxonase/arylesterase 1	1	16	5	Kumar,2012
Apolipoprotein E	1	16	5	Kumar,2012
Corticosteroid-binding globulin	1	16	5	Kumar,2012
Alpha-1-acid glycoprotein 2	1	16	5	Kumar,2012
Haptoglobin-related protein	1	16	5	Kumar,2012
Clusterin	1	16	5	Kumar,2012
Alpha-1-antitrypsin**	1	16	5	Kumar,2012
Apoliprotein CI	1	16	5	Kumar,2012

Apoliprotein CII	1	16	5	Kumar,2012
Apoliprotein CIII	1	16	5	Kumar,2012
Apoliprotein CIV	1	16	5	Kumar,2012
Platelet basic protein	1	16	5	Kumar,2012
FT	1	16	5	Kumar,2012
A2M	1	16	5	Kumar,2012
Endocan	1	16	5	Lam,2020
Endoglin	1	15	5	Mariappan,2021
MCP-2 (CCL8)	1	17	5	Pang,2016
MIP1a (CCL3)	1	17	5	Pang,2016
IL-2	1	17	5	Pang,2016
IFN-a2	1	17	5	Pang,2016
TRAIL(TNFSF10)	1	17	5	Pang,2016
Fibrinogen (FGA)	1	17	5	Pang,2016
Urea	1	16	5	Sigera, 2018
Creatinine	1	16	5	Sigera, 2018
Sodium	1	16	5	Sigera, 2018
Potassium	1	16	5	Sigera, 2018
Heparan sulfate	1	17	5	Suwarti,2017
VE-cadherin	1	17	5	Suwarti,2017
25(OH)D	1	15	5	Villamor,2017
VDBD	1	15	5	Villamor,2017
14 : 0 myristic acid	1	16	5	Villamore,2018
17 : 0 margaric acid	1	16	5	Villamore,2018
20 : 0 arachidic acid	1	16	5	Villamore,2018
24 : 0 lignoceric acid	1	16	5	Villamore,2018
16 : 1n-7 palmitoleic acid	1	16	5	Villamore,2018
18 : 1n-9 oleic acid	1	16	5	Villamore,2018
20 : 1n-9 gondoic acid	1	16	5	Villamore,2018
24 : 1n-9 nervonic acid	1	16	5	Villamore,2018

Total MUFA	1	16	5	Villamore,2018
18 : 3n-3 α-linolenic acid	1	16	5	Villamore,2018
20 : 5n-3 EPA	1	16	5	Villamore,2018
18 : 2n-6 linoleic acid	1	16	5	Villamore,2018
18 : 3n-6 γ-linolenic acid	1	16	5	Villamore,2018
2n-6EDA	1	16	5	Villamore,2018
Total n-6 PUFA	1	16	5	Villamore,2018
18 : 2n-7ct CLA (%)	1	16	5	Villamore,2018
16 : 1n-7 trans	1	16	5	Villamore,2018
18 : 1 trans	1	16	5	Villamore,2018
18 : 2 trans	1	16	5	Villamore,2018
Total trans-FA	1	16	5	Villamore,2018
Stearoyl-coA-desaturase 18 : 1n-9/18 : 0	1	16	5	Villamore,2018
Elongase 18 : 1n-7/16 : 1n-7	1	16	5	Villamore,2018
Δ6-Desaturase 18 : 3n-6/18 : 2n-6	1	16	5	Villamore,2018
Δ5-Desaturase 20 : 4n-6/20 : 3n-6	1	16	5	Villamore,2018
Arginase	1	15	5	Yacoub,2017
L-arginine	1	15	5	Yacoub,2017
Chymase	1	16	5	Rathore,2020
CH50	1	14	6	Yamanaka,2013
C1q	1	14	6	Yamanaka,2013
Fb	1	14	6	Yamanaka,2013
FI	1	14	6	Yamanaka,2013
fH	1	14	6	Yamanaka,2013
GGT	1	10	6	Fernando, 2016
PTX-3	1	13	6	Low, 2018
MCP-1	2	17	7	Kumar,2012
Calcium	2	16	7	Sigera, 2018
ferritin	177	14	7	Chaiyaratana,2008
Amylase	2	16	7	Sigera,2019

albumin 2009(controversial)	3	16	8	Fernando,2016
ALT	5	16	8	Fernando,2016
Ang-1	2	16	8	Mapalagamage, ,2020
Cholesterol	2	15	8	Biswas,2015
FactorD	2	16	8	Conoray,2015
FGFBASIC	2	16	8	Cui,2016
HDL	2	15	8	Biswas,2015
IL-1b	2	16	8	Cui,2016
IL-4	2	16	8	Cui,2016
IL-6	2	16	8	Villamor,2017
IL-10	582	16	8	Villamor,2017
LDL	2	15	8	Villar Centeno,2008
RANTES	2	16	8	Cui,2016
VEGF	2	16	8	Conoray,2015
CPK	3	16	8	Sigera,2018
G-CSF	2	16	8	Cui,2016
IFN-g	3	16	8	Cui,2016
IL-1ra	2	16	8	Kumar,2012
MIP1B	2	16	8	Cui,2016
TNF-a	2	16	8	Cui,2016
ALP	2	16	8	Sigera, 2018, Fernando 2016
IL-17	2	16	8	Kumar,2012

*Biomarker ranking categories:

Category 1 – confirmed as statistically significant in a meta-analysis

Category 2 – confirmed as statistically significant in two or more studies that cannot be combined in a meta-analysis due to inadequate data reporting, differences in units of measurement or other reasons

Category 3 – confirmed as statistically significant in one tier 1 study

Category 4 – confirmed as statistically significant in one tier 2 study

Category 5 – confirmed as statistically insignificant in one tier 2 study

Category 6 - confirmed as statistically insignificant in one tier 1 study

Category 7 - confirmed as statistically insignificant in two or more studies that cannot be combined in a meta-analysis

Category 8 - confirmed as statistically insignificant in a meta-analysis

When a single biomarker had two rankings (depending on the outcome considered – DHF or severe dengue), the higher ranking is shown in the table.

PRISMA 2020 Checklist

Section and Topic	Item #	Checklist item	Location where item is reported
**TITLE**			
Title	1	Identify the report as a systematic review.	1
**ABSTRACT**			
Abstract	2	See the PRISMA 2020 for Abstracts checklist.	2
**INTRODUCTION**			
Rationale	3	Describe the rationale for the review in the context of existing knowledge.	3
Objectives	4	Provide an explicit statement of the objective(s) or question(s) the review addresses.	4
**METHODS**			
Eligibility criteria	5	Specify the inclusion and exclusion criteria for the review and how studies were grouped for the syntheses.	4,5
Information sources	6	Specify all databases, registers, websites, organisations, reference lists and other sources searched or consulted to identify studies. Specify the date when each source was last searched or consulted.	5
Search strategy	7	Present the full search strategies for all databases, registers and websites, including any filters and limits used.	Table 1
Selection process	8	Specify the methods used to decide whether a study met the inclusion criteria of the review, including how many reviewers screened each record and each report retrieved, whether they worked independently, and if applicable, details of automation tools used in the process.	5,6,supplementary File 2
Data collection process	9	Specify the methods used to collect data from reports, including how many reviewers collected data from each report, whether they worked independently, any processes for obtaining or confirming data from study investigators, and if applicable, details of automation tools used in the process.	6
Data items	10a	List and define all outcomes for which data were sought. Specify whether all results that were compatible with each outcome domain in each study were sought (e.g. for all measures, time points, analyses), and if not, the methods used to decide which results to collect.	6
	10b	List and define all other variables for which data were sought (e.g. participant and intervention characteristics, funding sources). Describe any assumptions made about any missing or unclear information.	6
Study risk of bias assessment	11	Specify the methods used to assess risk of bias in the included studies, including details of the tool(s) used, how many reviewers assessed each study and whether they worked independently, and if applicable, details of automation tools used in the process.	6,7
Effect measures	12	Specify for each outcome the effect measure(s) (e.g. risk ratio, mean difference) used in the synthesis or presentation of results.	6,7
Synthesis methods	13a	Describe the processes used to decide which studies were eligible for each synthesis (e.g. tabulating the study intervention characteristics and comparing against the planned groups for each synthesis (item #5)).	6
	13b	Describe any methods required to prepare the data for presentation or synthesis, such as handling of missing summary statistics, or data conversions.	6
	13c	Describe any methods used to tabulate or visually display results of individual studies and syntheses.	7
	13d	Describe any methods used to synthesize results and provide a rationale for the choice(s). If meta-analysis was performed, describe the model(s), method(s) to identify the presence and extent of statistical heterogeneity, and software package(s) used.	7
	13e	Describe any methods used to explore possible causes of heterogeneity among study results (e.g. subgroup analysis, meta-regression).	7
	13f	Describe any sensitivity analyses conducted to assess robustness of the synthesized results.	7
Reporting bias assessment	14	Describe any methods used to assess risk of bias due to missing results in a synthesis (arising from reporting biases).	8
Certainty assessment	15	Describe any methods used to assess certainty (or confidence) in the body of evidence for an outcome.	7

PRISMA 2020 Checklist

Section and Topic	Item #	Checklist item	Location where item is reported
**RESULTS**			
Study selection	16a	Describe the results of the search and selection process, from the number of records identified in the search to the number of studies included in the review, ideally using a flow diagram.	8
	16b	Cite studies that might appear to meet the inclusion criteria, but which were excluded, and explain why they were excluded.	8
Study characteristics	17	Cite each included study and present its characteristics.	8
Risk of bias in studies	18	Present assessments of risk of bias for each included study.	8,9, supplementary Table 4
Results of individual studies	19	For all outcomes, present, for each study: (a) summary statistics for each group (where appropriate) and (b) an effect estimate and its precision (e.g. confidence/credible interval), ideally using structured tables or plots.	9 – 11, Figures 2-4
Results of syntheses	20a	For each synthesis, briefly summarise the characteristics and risk of bias among contributing studies.	9,10,11
	20b	Present results of all statistical syntheses conducted. If meta-analysis was done, present for each the summary estimate and its precision (e.g. confidence/credible interval) and measures of statistical heterogeneity. If comparing groups, describe the direction of the effect.	9,10,11
	20c	Present results of all investigations of possible causes of heterogeneity among study results.	12. 13
	20d	Present results of all sensitivity analyses conducted to assess the robustness of the synthesized results.	11,12
Reporting biases	21	Present assessments of risk of bias due to missing results (arising from reporting biases) for each synthesis assessed.	N/A
Certainty of evidence	22	Present assessments of certainty (or confidence) in the body of evidence for each outcome assessed.	13, Supplementary file 3
**DISCUSSION**			
Discussion	23a	Provide a general interpretation of the results in the context of other evidence.	12
	23b	Discuss any limitations of the evidence included in the review.	15
	23c	Discuss any limitations of the review processes used.	16
	23d	Discuss implications of the results for practice, policy, and future research.	18
**OTHER INFORMATION**			
Registration and protocol	24a	Provide registration information for the review, including register name and registration number, or state that the review was not registered.	2,7
	24b	Indicate where the review protocol can be accessed, or state that a protocol was not prepared.	7
	24c	Describe and explain any amendments to information provided at registration or in the protocol.	19
Support	25	Describe sources of financial or non-financial support for the review, and the role of the funders or sponsors in the review.	Submission system
Competing interests	26	Declare any competing interests of review authors.	Submission system
Availability of data, code and other materials	27	Report which of the following are publicly available and where they can be found: template data collection forms; data extracted from included studies; data used for all analyses; analytic code; any other materials used in the review.	Supplementary File 1

PRISMA 2020 Checklist

From: Page MJ, McKenzie JE, Bossuyt PM, Boutron I, Hoffmann TC, Mulrow CD, et al. The PRISMA 2020 statement: an updated guideline for reporting systematic reviews. BMJ 2021;372:n71. doi: 10.1136/bmj.n71
For more information, visit: http://www.prisma-statement.org/
